# Early effects of oral administration of lafutidine with mosapride compared with lafutidine alone on intragastric pH values

**DOI:** 10.1186/1471-230X-9-52

**Published:** 2009-07-09

**Authors:** Hiroshi Iida, Masahiko Inamori, Yuichi Nozaki, Hiroki Endo, Kunihiro Hosono, Tomoyuki Akiyama, Yasunari Sakamoto, Hirokazu Takahashi, Tomoko Koide, Chikako Tokoro, Yasunobu Abe, Atsushi Nakajima

**Affiliations:** 1 Gastroenterology Division, Yokohama City University School of Medicine, 3-9 Fukuura, Kanazawa-ku, Yokohama, Japan

## Abstract

**Background:**

The ideal medication for treatment of acid related diseases should have a rapid onset of action to promote hemostasis and resolution of symptoms. The aim of our study was to investigate the inhibitory effects on gastric acid secretion after a single oral administrations of lafutidine, is a newly synthesized H2-receptor antagonist, with mosapride 5 mg or lafutidine alone.

**Methods:**

Ten *Helicobacter pylori *negative male subjects participated in this randomized, two-way crossover study. Intragastric pH was monitored continuously for 4 hours after a single oral administration of lafutidine 10 mg or lafutidine 10 mg with mosapride 5 mg (the lafutidine being administrated one hour after the mosapride). Each administration was separated by a 7-day washout period.

**Results:**

The average pH during the 4-hour period after administration of lafutidine 10 mg with mosapride 5 mg was higher than after lafutidine 10 mg alone (median: 5.25 versus 4.58, respectively; *p *= 0.0318). During the 3–4 hour study period, lafutidine 10 mg with mosapride 5 mg provided a higher pH, compared to lafutidine 10 mg alone (median: 7.28 versus 6.42; *p *= 0.0208).

**Conclusion:**

In *H. pylori *negative healthy male subjects, an oral dose of lafutidine 10 mg with mosapride 5 mg more rapidly increased intragastric pH than lafutidine 10 mg alone.

## Background

The ideal medication for gastric acid related diseases, especially for example hemorrhagic gastric ulcers and stress related gastric bleeding, should have a rapid onset of action to lower intragastric acidity, because *in vitro *studies have shown that blood coagulation and platelet aggregation are abolished at a pH less than 5.4 [1]. Medication for on-demand treatment also should have a rapid onset of action to assure that symptoms are controlled. Multiple agents including antacids, histamine H2 receptor antagonists (H2RAs) and proton pump inhibitors (PPIs) are currently available. PPIs are the most potent inhibitors of gastric acid secretion when used regularly [2]. However, no study has yet examined whether an H2RA with mosapride (orally ingested) more rapidly increases intragastric pH than the H2RA on its own. This crossover study was designed to compare the acute effect on the intragastric pH following administration of lafutidine 10 mg on its own, or lafutidine 10 mg with mosapride 5 mg.

## Methods

### Subjects

This was a randomized, two-way crossover study with 10 healthy male volunteers, mean age 27.1 years (range 21 – 35 years), who were not users of acid suppressive medications including antacids, H2RAs, and/or PPIs. All subjects were negative for anti-*Helicobacter pylori *(*H. pylori*) immunoglobulin G antibodies (SRL Inc, Tokyo, Japan).

### Study Protocol and pH-metry

All subjects followed two study protocols in which they were given 10 mg lafutidine (Protecadin, Taiho Pharmaceutical Co. Ltd, Tokyo, Japan) *per os*, or 10 mg lafutidine and 5 mg mosapride (Gasmotin, Dainippon Sumitomo pharmaceutical Co. Ltd, Osaka, Japan) with the lafutidine being administrated one hour after the mosapride. Intragastric pH was monitored continuously for 4 hours after each protocol. There was a washout period of at least 7 days between each protocol. The subjects fasted overnight (at least 8 hours) before each treatment protocol and during the 4 hours after ingesting the drug or drugs, and both experiments were performed in the morning.

The pH electrode was inserted transnasally under local anaesthesia and located in the body of the stomach. The gastric pH was measured at 10 second intervals by a portable pH meter attached to the antimony pH electrode (Chemical Instrument Co. Ltd, Tokyo, Japan). The pH electrode was calibrated before each recording using standard buffers of pH 4.01 and 6.86. The pH data were analyzed using established software (Chemical Instrument Co. Ltd, Tokyo, Japan). The average pH and the percentage of times during which intragastric pH remained above 1, 2, 3, 3.5, 4, 5, 6, 7 and 8 over the 4 hour monitoring period after the administration of each drug programme were also measured.

### Statistics

Statistical evaluation was carried out using the Wilcoxon signed-ranks test. The level of significance was p < 0.05. Statistical analyses were performed using the Stat View program (SAS Institute, Cary, NC, USA).

### Ethics

The study was conducted in accordance with the Declaration of Helsinki, and the Ethics Committee of Yokohama City University School of Medicine approved this study. We obtained written informed consent from all volunteers.

## Results

All subjects completed the study. No adverse events were recorded during the study.

### Average pH

**Figure 1:**
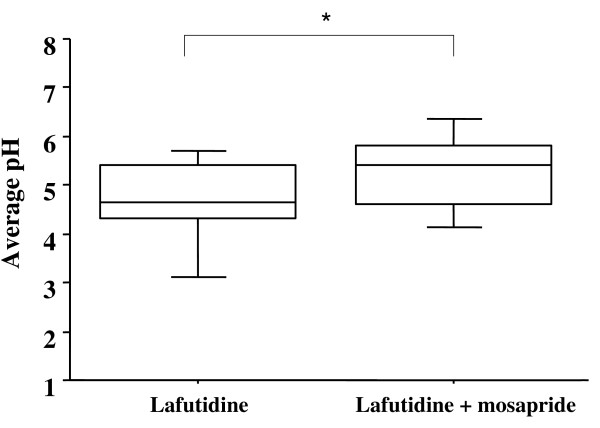
**Average pH during the first 4 hours was higher after administration of lafutidine with mosapride than after lafutidine**. *p = 0.0318 by the Wilcoxon signed-ranks test.

The average pH during the 4-hour period after the administration of lafutidine 10 mg with mosapride 5 mg was higher than after lafutidine 10 mg alone (median: 5.25 versus 4.58, respectively; *p *= 0.0318) (Figure 1).

**Figure 2:**
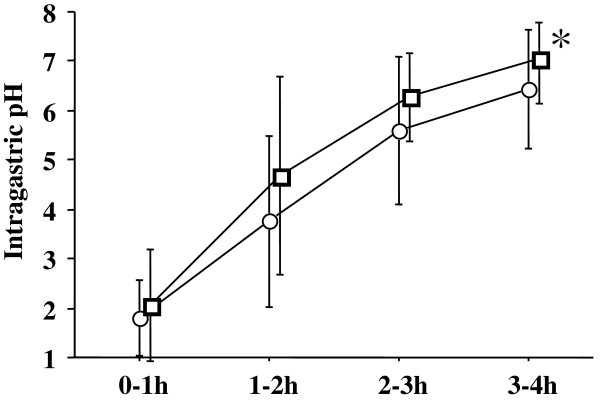
**Lafutidine 10 mg with mosapride 5 mg provided a higher average pH, compared to lafutidine 10 mg alone, at the 3–4 hour study period after administration**. Squares (lafutidine with mosapride) and circles (lafutidine), mean values; vertical lines, standard deviation (SD); horizontal line, ± SD. *p = 0.0208 by the Wilcoxon signed-ranks test.

The average pH was significantly higher after administration of lafutidine 10 mg with mosapride 5 mg, compared to lafutidine 10 mg on its own during the 3–4 hour study period (median: 7.28 versus 6.42; p = 0.0208) (Figure 2). No significant differences were found at the 0–1, 1–2 and 2–3 hour study periods.

### Holding time (%) of various pH levels over 4 hours

**Figure 3:**
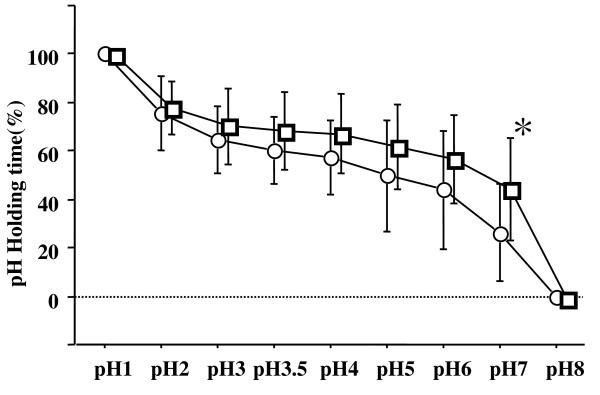
**During the 4-hour study period, lafutidine 10 mg with mosapride 5 mg provided a longer duration of pH > 7, compared to lafutidine 10 mg alone**. Squares (lafutidine with mosapride) and circles (lafutidine), mean values; vertical lines, standard deviation (SD); horizontal line, ± SD. *p = 0.0284 by the Wilcoxon signed-ranks test.

During the 4-hour study period, lafutidine 10 mg with mosapride 5 mg provided longer durations of pH above 2, 3, 3.5, 4, 5, 6 and 7 than did lafutidine 10 mg alone (median: 78.7% versus 75.6%; p = 0.5751, 70.8% versus 64.8%; p = 0.5071, 68.7% versus 59.9%; p = 0.1141, 67.6% versus 57.5%; p = 0.0593, 62.5% versus 49.8%; p = 0.1394, 57.4% versus 43.9%; p = 0.1688, and 44.4% versus 26.1%; p = 0.0284; respectovely) (Figure 3).

## Discussion

In this study, we examined the change of intragastric pH after a single oral administration of lafutidine 10 mg with mosapride 5 mg compared with lafutidine 10 mg alone in the early post-administration phase in *H. pylori *negative subjects.

Mosapride citrate (mosapride) (4-amino-5-chloro-2-ethoxy-N-{[4-(4-fluorobenzyl)-2-morpholinyl]methyl} benzamide citrate) is a novel gastrokinetic agent that enhances gastrointestinal motility by stimulating the serotonin (5-HT4) receptor [3]. Mosapride stimulates acetylcholine release from cholinergic neurons in the gastrointestinal wall and may enhance upper gastrointestinal motor activity in the postprandial state in conscious dogs [4]. After oral administration, mosapride is absorbed in the small intestine in rats, rather than the stomach [5].

Mosapride accelerates gastric emptying in healthy adults. This study may indicate that mosapride accelerates the absorption of lafutidine. For example, mosapride accelerates the gastric emptying and completion rate of small bowel examinations in patients undergoing capsule endoscopy [6]. The time of gastric emptying time (GET) in the mosapride group was reduced and indicated the obvious effect of mosapride on shortening GET. Moreover, Preparation for barium enemas using mosapride before and after oral intestinal lavage solution (PEG-ELS) intake is more effective than the modified Brown's method that is commonly used in Japan [7]. Mosapride improves gastrointestinal motility and reduces gastric stasis or gastroesophageal reflux [8,9]. Mosapride also alleviates gastrointestinal dysfunction.

Although many factors are implicated in the development of gastroesophageal reflux disease (GERD), acid reflux to the esophagus is considered to be the major causes of this disease. Treatment with PPIs to provide potent, long-term suppression of gastric acid is essential for disease management. On the other hand, the transient heartburn associated with mild GERD is attributed mainly to temporary, short-term gastric acid reflux. For example, water and antacid immediately increased gastric pH, while PPIs showed a delayed but prolonged effect compared to H2RAs [10].

Because lafutidine promptly suppressed gastric acid secrerion [11], it was orginally considered to be a useful drug for the on-demand treatment of mild GERD. Because mosapride accelerates gastric emptying, Lafutidine can pass from stomach to duodenum and be absorbed quickly. Lafutidine used with mosapride may cause the rapid onset of action, and may thus be more suitable for on-demand use.

The limitation of the present study is that the data were collected from healthy volunteers and not GERD patients requiring on-demand therapy. The clinical implications of our results remain unclear; however, our findings suggest that the orally administered combination of lafutidine 10 mg preceded by mosapride 5 mg tablets may be suitable for on-demand treatment in patients with GERD. Further studies are necessary to confirm this assumption.

## Conclusion

In *H. pylori *negative healthy male subjects, an oral dose of lafutidine 10 mg with mosapride 5 mg more rapidly increased intragastric pH than lafutidine 10 mg alone.

## List of abbreviations

H2RAs: histamine H2 receptor antagonists; PPIs: proton pump inhibitors; 5-HT4: 5-Hydroxytryptamine receptor 4; GET: gastric emptying time; PEG-ELS: polyethylene glycol electrolyte; GERD: gastroesophageal reflux disease

## Competing interests

The authors declare that they have no competing interests.

## Authors' contributions

HI analyzed, collected the clinical data and wrote the manuscript, with contributions from MI, CT an YN. AN, HT, TK and YA was responsible for the design of the study and collected the clinical data. MI, HE and KH performed the statistical analyses. HI, YS and MI analyzed the clinical data and participated in the design and coordination of the study. All authors read and approved the final manuscript.

## Pre-publication history

The pre-publication history for this paper can be accessed here:

http://www.biomedcentral.com/1471-230X/9/52/prepub
